# Supplementary material for: Modeling for strain-softening rocks with lateral damage based on statistical physics
Source: PLoS One. 2023 Mar 30;18(3):e0283313. doi: 10.1371/journal.pone.0283313 (PMC10062584; doi:10.1371/journal.pone.0283313)
Supplement: Attachment — Submitted filename: PONE-D-22-33973.pdf [file pone.0283313.s003.pdf]

PLOS ONE

Modeling for strain-softening rocks with lateral damage based on statistical physics

--Manuscript Draft--

**Manuscript Number:**	PONE-D-22-33973
**Article Type:**	Research Article
**Full Title:**	Modeling for strain-softening rocks with lateral damage based on statistical physics
**Short Title:**	Modeling for strain-softening rocks
**Corresponding Author:**	Mingwu Wang, Ph.D.<br>Hefei University of Technology<br>Hefei , CHINA
**Keywords:**	probability statistical; maximum entropy theory; lateral damage; constitutive model; Residual strength.
**Abstract:**	Statistical physics is widely used to study the nonlinear mechanical behaviors of rock. For the limitations of existing statistical damage models and Weibull distribution, a new statistical damage with lateral damage is established. In addition, by introducing the maximum entropy distribution function and the strict constraint on damage variable, a expression of the damage variable matching the proposed model is obtained. Through comparing with the experimental results and the Weibull statistical damage model, the rationality of the maximum entropy statistical damage model is confirmed. The proposed model can better reflect the strain-softening behavior for rocks and respond to the residual strength, which provides a theoretical reference for practical engineering construction and design.
**Order of Authors:**	Xiaoming Li
	Mingwu Wang, Ph.D.
	Fengqiang Shen
	Hongfei Zhang
**Additional Information:**	
**Question**	**Response**
**Financial Disclosure**<br><br>Enter a financial disclosure statement that describes the sources of funding for the work included in this submission. Review the submission guidelines for detailed requirements. View published research articles from *PLOS ONE* for specific examples.<br><br>This statement is required for submission and **will appear in the published article** if the submission is accepted. Please make sure it is accurate.	This work was supported by the National Natural Sciences Foundation of China (No 41172274) recipient: Mingwu Wang

Powered by Editorial Manager® and ProduXion Manager® from Aries Systems Corporation

**Unfunded studies**<br>Enter: *The author(s) received no specific funding for this work.*<br><br>**Funded studies**<br>Enter a statement with the following details:<br>• Initials of the authors who received each award<br>• Grant numbers awarded to each author<br>• The full name of each funder<br>• URL of each funder website<br>• Did the sponsors or funders play any role in the study design, data collection and analysis, decision to publish, or preparation of the manuscript?<br>• **NO** - Include this sentence at the end of your statement: *The funders had no role in study design, data collection and analysis, decision to publish, or preparation of the manuscript.*<br>• **YES** - Specify the role(s) played.<br><br>* typeset	
**Competing Interests**<br><br>Use the instructions below to enter a competing interest statement for this submission. On behalf of all authors, disclose any competing interests that could be perceived to bias this work—acknowledging all financial support and any other relevant financial or non-financial competing interests.<br><br>This statement is required for submission and **will appear in the published article** if the submission is accepted. Please make sure it is accurate and that any funding sources listed in your Funding Information later in the submission form are also declared in your Financial Disclosure statement.<br><br>View published research articles from *PLOS ONE* for specific examples.	The authors have declared that no competing interests exist

**NO authors have competing interests**<br><br>Enter: *The authors have declared that no competing interests exist.*<br><br>**Authors with competing interests**<br><br>Enter competing interest details beginning with this statement:<br><br>*I have read the journal's policy and the authors of this manuscript have the following competing interests: [insert competing interests here]*<br><br>* typeset	
**Ethics Statement**<br><br>Enter an ethics statement for this submission. This statement is required if the study involved:<br><br>• Human participants<br>• Human specimens or tissue<br>• Vertebrate animals or cephalopods<br>• Vertebrate embryos or tissues<br>• Field research<br><br>Write "N/A" if the submission does not require an ethics statement.<br><br>General guidance is provided below. Consult the submission guidelines for detailed instructions. **Make sure that all information entered here is included in the Methods section of the manuscript.**	N/A

Powered by Editorial Manager® and ProduXion Manager® from Aries Systems Corporation

**Format for specific study types**<br><br>**Human Subject Research (involving human participants and/or tissue)**<br>• Give the name of the institutional review board or ethics committee that approved the study<br>• Include the approval number and/or a statement indicating approval of this research<br>• Indicate the form of consent obtained (written/oral) or the reason that consent was not obtained (e.g. the data were analyzed anonymously)<br><br>**Animal Research (involving vertebrate animals, embryos or tissues)**<br>• Provide the name of the Institutional Animal Care and Use Committee (IACUC) or other relevant ethics board that reviewed the study protocol, and indicate whether they approved this research or granted a formal waiver of ethical approval<br>• Include an approval number if one was obtained<br>• If the study involved *non-human primates*, add *additional details* about animal welfare and steps taken to ameliorate suffering<br>• If anesthesia, euthanasia, or any kind of animal sacrifice is part of the study, include briefly which substances and/or methods were applied<br><br>**Field Research**<br><br>Include the following details if this study involves the collection of plant, animal, or other materials from a natural setting:<br>• Field permit number<br>• Name of the institution or relevant body that granted permission	
**Data Availability**<br><br>Authors are required to make all data underlying the findings described fully available, without restriction, and from the time of publication. PLOS allows rare exceptions to address legal and ethical concerns. See the PLOS Data Policy and FAQ for detailed information.	Yes - all data are fully available without restriction

A Data Availability Statement describing where the data can be found is required at submission. Your answers to this question constitute the Data Availability Statement and **will be published in the article**, if accepted.<br><br>**Important:** Stating 'data available on request from the author' is not sufficient. If your data are only available upon request, select 'No' for the first question and explain your exceptional situation in the text box.<br><br>Do the authors confirm that all data underlying the findings described in their manuscript are fully available without restriction?	
**Describe where the data may be found in full sentences. If you are copying our sample text, replace any instances of XXX with the appropriate details.**<br><br>• If the data are **held or will be held in a public repository**, include URLs, accession numbers or DOIs. If this information will only be available after acceptance, indicate this by ticking the box below. For example: *All XXX files are available from the XXX database (accession number(s) XXX, XXX.)*.<br>• If the data are all contained **within the manuscript and/or Supporting Information files**, enter the following: *All relevant data are within the manuscript and its Supporting Information files.*<br>• If neither of these applies but you are able to provide **details of access elsewhere**, with or without limitations, please do so. For example:<br><br>*Data cannot be shared publicly because of [XXX]. Data are available from the XXX Institutional Data Access / Ethics Committee (contact via XXX) for researchers who meet the criteria for access to confidential data.*<br><br>*The data underlying the results presented in the study are available from (include the name of the third party*	All relevant data are within the manuscript and its Supporting Information files.

and contact information or URL).<br>• This text is appropriate if the data are owned by a third party and authors do not have permission to share the data.<br><br>* typeset	
Additional data availability information:	

Manuscript Click here to access/download;Manuscript;Manuscript.docx

Modeling for strain-softening rocks with lateral damage based on statistical physics

Xiaoming Li [1,2], Mingwu Wang [1,*], Fengqiang Shen [1], Hongfei Zhang[3]

[1]School of Civil and Hydraulic Engineering, Hefei University of Technology, Hefei , China

[2]School of City and Architecture Engineering, Zaozhuang University, Zaozhuang , China

[3]Shandong Zhengyuan Geological Resources Exploration co.ltd, Jinan , China

*Corresponding author

E-mail: wanglab307@foxmail.com (Mingwu Wang)

Abstract

Statistical physics is widely used to study the nonlinear mechanical behaviors of rock. For the limitations of existing statistical damage models and Weibull distribution, a new statistical damage with lateral damage is established. In addition, by introducing the maximum entropy distribution function and the strict constraint on damage variable, a expression of the damage variable matching the proposed model is obtained. Through comparing with the experimental results and the Weibull statistical damage model, the rationality of the maximum entropy statistical damage model is confirmed. The proposed model can better reflect the strain-softening behavior for rocks and respond to the residual strength, which provides a theoretical reference for practical engineering construction and design.

Keywords： probability statistical; maximum entropy theory; lateral damage; constitutive model; Residual strength.

Introduction

Please give a reference or expalin how its more complex than other materials.

Unlike most continuous materials, rock is a complex geological material, and its mechanical progressive failure behavior is also very intricate under external loads, with strong nonlinear characteristics. Since the concept of the stress-strain relationship of whole process for rocks was proposed [1], the study of rock constitutive model that reflects the stress-strain relationship of whole process has been the focus of traditional rock mechanics [2–7].

The complexity of rocks failure mechanism results in a strong nonlinear stress-strain relationship. Krajcinovic [8] firstly introduced statistics physics to study the stress-strain relationship of whole process for rocks, to some extent, which reveals the correlation mechanism between macroscopic phenomenology and mesoscopic damage.Then, some academics have done continuous researches on the damage evolution characteristics of rocks by using statistics and obtained beneficial results [9–14]. To reflect rocks' strain hardening or softening behaviors, combining statistical theory with continuous damage theory, Xu [15] proposed a statistical damage model. Still, it cannot show the residual strength of rocks. Considering that it is difficult for existing models to explain the residual strength, Xu [16] and Zhu [17] improved the damage mode by introducing a damage correction factor. The development of rock damage is a slow process of damage accumulation, and the undamaged area would gradually change into the damaged area. The damaged area would still bear the load rather than become a hollow area [18-20]. In recent years, scholars began to consider various factors to establish more scientific and reasonable statistical damage constitutive models. Considering that rocks can't (cannot) be damaged under low levels of stress, Jiang [21] introduced a damage threshold into the model to control the occurrence of the rock damage.

Lin [22] suggested that the size effect should be considered in studying the failure process of rock with mesoscopic damage mechanics. Most of the above models still belong to the traditional elastic-plastic model, in which continuous damage mechanics were modified, and the damage variables were obtained by statistics theory. But there are still some problems with these models and need further study.

One problem, there is no doubt, so far, the most widely used probability density distribution function is the Weibull distribution. However, Weibull distribution has its limitations and does not work in some cases. For example, the number of samples should not be less than 30 [23], the materials of rock with complex defect density or brittle [24,25], and the probability distribution of rock strength is not approximate to the power low [26]. By contrast, the maximum entropy distribution function can overcome these problems [27,28]. As a non-parametric probability density distribution estimation method, the maximum entropy theory can directly infer the distribution function of the parameter variables based on the information of test samples and statistical methods without assuming the distribution of the parameter variables in advance. Besides, information entropy [29] reflects the randomness of the parameter, so it is scientifically reasonable to infer the distribution function of the randomly distributed parameter variables by using the maximum entropy theory. Meanwhile, it can avoid too much additional personal information. Unfortunately, the maximum entropy distribution function is rarely used to study the stress-strain behavior of rock, although Deng [5] proved the feasibility that the maximum entropy distribution function can describe rock mechanical behaviors, there is no reasonable application constraints, so there is a risk that the calculated value of damage variable may overflow ($D \geq 1$), which is inconsistent with the

assumption of damage variable.

Another problem is that existing statistical constitutive models [3,5,10,16,17-20] for rock tend to focus on axial damage but often ignore lateral damage, which is not very reasonable and inconsistent with the actual situation.

Focusing on the above problems, this paper assumed that the strength of rock meso-element obeys the maximum entropy distribution, and which specific probability distribution function is deduced, then the damage variable is obtained according to the statistical damage theory of rock. Furthermore, based on the meso-damage statistics theory, a new statistical constitutive model considering the lateral damage is proposed, and its constitutive relation equation is deduced. Finally, using experimental data, the proposed model is verified by comparing the Weibull model. This study provides some reference significance for the study of the stress-strain whole process for rock materials.

Methodology

The maximum entropy principle

Information entropy is used to reflect the amount of information transferred among systems. The larger the information entropy is, the less information transmitted between systems will be; the higher the uncertainty, the greater the randomness, and vice versa. Therefore, there is a specific relationship between information entropy and the randomness of events. Let x be the random variable, and $f(x)$ is the continuous probability density distribution for the random variable x, then its information entropy can be expressed as [5]

$$H(x) = E[-\ln(f(x)] = -\int_R f(x)\ln(f(x))dx \tag{1}$$

Where $H(x)$ is the information entropy; E represents the mathematical expectation; R denotes

the range of *x*.

For a particular sample group, Jaynes [27,28] holds that *f*(*x*) with the maximum information entropy is the most unbiased under certain information conditions, then Eq. (1) can be written as

$$H(x)_{\max} = E\left[-\ln(f_p(x)\right] = -\int_R f_p(x)\ln(f_p(x))dx \tag{2}$$

where $H(x)_{\max}$ represents the maximum information entropy of x; $f_p(x)$ is the probability density function corresponding to $H(x)_{\max}$. Just like the traditional rock statistical constitutive model, assuming that the strength of the rock meso-element is *x*, $f_p(x)$ can be obtained by solving Eq. (2) when $H(x)$ is at the maximum value. Because the maximum value of the damage variable is 1, $f_p(x)$ needs to be constrained as

$$\int_R f_p(x) = 1 \tag{3}$$

$f_p(x)$ cannot be directly solved by Eqs. (2) and (3) need to transform the direct solution problem into an optimal problem by adding constraints. Herein, the information entropy constraints mainly consist of the characteristics of the probability distribution and the statistical characteristics of the sample data. All in all, the optimal problem can be formulated as

$$\begin{cases} f_p(x) \in \arg\max H(x) = -\int_R f_p(x)\ln(f_p(x))dx \\ \quad s.t. \ \int_R f_p(x) = 1 \\ \quad\quad \int_R g_i(x) f_p(x)dx = b_i \end{cases} \tag{4}$$

Where $g_i(x)$ represents the restriction function. $g_i(x)$ has an indefinite form. Deng [5] suggested its form is x^i (i=0, 1, …, m). b_i is the original moment of samples. To solve Eq. (4), Eq. (1) should be transformed into a Lagrange function as

$$H(x) = -\int_R f(x)\ln f(x)\mathrm{d}x - \lambda_0\int_R (f(x)dx - 1) + \sum_{i=1}^{m}\lambda_i\left[\int_R g_i(x) f(x)dx\right] - b_i \tag{5}$$

Where λ_i and λ_0 represent the Lagrange multiplier. If $H(x)$ is at the maximum value, the derivative of Eq. (5) is required to be zero, and the result is shown as

$$\frac{\partial H(x)}{\partial f(x)} = 0 \Rightarrow f_p(x) = \exp(-\lambda_0 - \sum_{i=1}^{m} \lambda_i g_i(x)) \tag{6}$$

Substituting Eq. (6) into the restriction function into Eq. (4), a nonlinear system of equations about the Lagrange multipliers is

$$\int_R \exp(-\lambda_0 - \sum_{i=1}^{m} \lambda_i g_i(x))dx = 1 \tag{7}$$

$$\int_R g_i(x)\exp(-\lambda_0 - \sum_{i=1}^{m} \lambda_i g_i(x)) = b_i \tag{8}$$

The Lagrange multipliers λ_0, λ_0, …, λ_i can be derived by solving Eqs. (7) and (8) with numerical solution. Next, the calculated values of λ_0, λ_0, …, λ_i are substituted into Eq. (6), the specific functional form of $f_p(x)$ is determined. According to the statistical definition, the probability distribution function of the meso-element strength for rocks is written as

$$F(x) = \int_0^R f_p(x)dx = \int_0^R \exp(-\lambda_0 - \sum_{i=1}^{m} \lambda_i g_i(x))dx \tag{9}$$

where F(x) is the probability distribution function of the meso-element strength.

Statistical damage evolution of rocks

Suppose that the macroscopic rock is made up of N meso-elements which are continuous with each other. As the stress level increases, these meso-elements are destroyed, and their number N_d increases, the damage variable D can be expressed as [10,18]

$$D = \frac{N_d}{N} = \frac{N \cdot F(x)}{N} = F(x) \tag{10}$$

with substituting Eq. (9) into Eq. (10), the damage variable with maximum entropy can be expressed as

$$D=\int_0^R \exp(-\lambda_0-\sum_{i=1}^{m}\lambda_i g_i(x))dx \tag{11}$$

For the convenience of subsequent expression, the expression symbol of the strength for rock meso-element is replaced by *F*, then Eq. (11) is written as

$$D=\int_0^R \exp(-\lambda_0-\sum_{i=1}^{m}\lambda_i g_i(F))dF \tag{12}$$

where *D* is the damage variable of rock with maximum entropy, *F* is the strength of rock meso-element.

Establishment of the statistical damage model

Model derivation

The damage variable will affect the constitutive relation of rock material, which can increase the effective stress or reduce the equivalent elastic modulus. The corresponding relationship can be characterized as follows [30]

$$\sigma^* = \sigma/1-D = E\varepsilon/1-D \tag{13}$$

where σ* is the effective stress; σ is the apparent stress; *E* is the elastic modulus; ε is strain; *D* is the damage variable.

Under the external loading, an element has two states of failure and non-failure [3,5,10,20] as shown in Fig 1. These correspond to the two states of imaginary rock damaged and undamaged, respectively. S_1^1 and S_1^2 represent the non-failure and the part failure part, respectively. S_2^1 and S_2^2 denotes the lateral non-failure part and the failure part, respectively. Then the axial damage variable can be expressed as:

please check this.

$$D=\frac{S_1^2}{S_1^1+S_1^2} \tag{14}$$

Fig 1. The mechanical analysis of rock meso-elements.

Move to the end with the corresponded figure.

As shown in Fig 1, σ_1^* and σ_1' is the axial net stress applied on the non-failure part and the failure part, respectively; σ_3^* and σ_3' is the lateral net stress involved on the non-failure part and the failure part, respectively. Here, let $\sigma_1' = \sigma_1^r$, σ_1^r is the axial residual strength, based on static equilibrium in the axial direction, we have

$$\sigma_1(S_1^1 + S_1^2) = \sigma_1^* S_1^1 + \sigma_1^r S_1^2 \tag{15}$$

where σ_1 is the axial apparent stress. Eq. (14) is substituted into Eq. (15), then Eq. (15) can be written as

$$\sigma_1 = \sigma_1^*(1\text{-}D) + \sigma_1^r D \tag{16}$$

In terms of lateral damage, considering that in triaxial test, the confining pressure remains unchanged, and when rock is completely damaged, the net stress in the damaged area should be equal to the confining pressure. Based on the above description, let $\sigma_3' = D\sigma_3$, namely, in the case of constant confining pressure, it is assumed that the net stress in the lateral damage area is proportional to the damage degree, and the lateral static equilibrium expression is written as follows

$$\sigma_3 = \sigma_3^*(1-D) + (\sigma_3 D).D \tag{17}$$

Eqs. (16) and (17) are different from the equations derived in existing studies that ignore the lateral damage or are replaced σ_3' with σ_1^r. By investigating Eq. (17), when the damage variable gradually rises to the maximum value (D=1), the lateral apparent stress value is equal to the confining pressure. It is consistent with the actual situation. Since the proposed damage mechanics model itself is a mathematical model hypothesis, there is no unified specific expression of the relationship between rock's exact mesoscopic physical damage mechanism and macroscopic phenomena. On the other hand, the primary purpose of the assumption of

net stress in the lateral damage area is to solve the problem of axial residual strength. Therefore, so the expression of the mechanical mechanism of the lateral hypothesis will not be discussed in depth.

According to the mechanics of materials, each principal stress produces a linear strain in two directions besides its own for a single element body. Let ε_1 be the principal strain in the direction of σ_1, the expression of ε_1 can be obtained as

$$\varepsilon_1 = \varepsilon_1' + \varepsilon_1'' + \varepsilon_1''' \tag{18}$$

where ε_1' represents the linear strain of σ_1 in the axial direction; ε_1'' is the linear strain of σ_2 in the axial direction; ε_1''' denotes the linear strain of σ_3 in the axial direction. Based on strain coordination, the strain produced by the undamaged material is consistent with that of the damaged material, thus

$$\varepsilon_i^* = \varepsilon_i^r \tag{19}$$

Combined with Eq. (19) and Hooke's law, the following physical equations can be obtained as

$$\begin{cases} \varepsilon_1' = \sigma_1^* / E \\ \varepsilon_1'' = -\mu\sigma_2^* / E \\ \varepsilon_1'' = -\mu\sigma_3^* / E \end{cases} \tag{20}$$

Substitute Eq. (20) into Eq. (18), the new expression of ε_1 is given as follows

$$\varepsilon_1 = \frac{\sigma_1^*}{E} - \frac{2\mu\sigma_3^*}{E} \tag{21}$$

that is

$$\sigma_1^* = \varepsilon_1 E + 2\mu\sigma_3^* \tag{22}$$

Then, substitute Eq. (22) into Eq. (16), the following equation is given

$$\sigma_1 = (E\varepsilon_1 + 2\mu\sigma_3^*)(1 - D) + \sigma_1^r D \tag{23}$$

Eq. (17) is substituted into Eq. (23), Eq. (23) can be further written as

$$\sigma_1 = E\varepsilon_1(1-D) + 2\mu\sigma_3(1-D^2) + \sigma_1^r D \quad (24)$$

In addition, it is well-known that rock damage is closely related to the stress level, so it is necessary to determine a damage threshold to control the onset of damage. The final statistical model proposed here can be obtained by substituting Eq. (12) into Eq. (24), we have

$$\begin{cases} \sigma_1 = E\varepsilon_1 + 2\mu\sigma_3 + (\sigma_1^r - E\varepsilon_1)\left(\int_0^R \exp(-\lambda_0 - \sum_{i=1}^{m} \lambda_i g_i(x))dx\right) - 2\mu\sigma_3\left[\int_0^R \exp(-\lambda_0 - \sum_{i=1}^{m} \lambda_i g_i(x))dx\right]^2 & \text{For } F > 0 \\ \sigma_1 = E\varepsilon_1 + 2\mu\sigma_3 & \text{For } F \le 0 \end{cases} \quad (25)$$

where F represents the strength of rock meso-element. For Equation (25), when $F \le 0$, namely no damage (D=0) occurs to rocks, the constitutive model of rock will degenerate to the traditional form.

Determination of the strength for meso-element

The strength values of the rock meso-elements are the basic for determining the probability density distribution function, which was defined in different ways. Cao [31] pointed out that the strength values (F) of rock meso-elements are a function of stress levels, internal friction angle, and cohesion. Pariseau [32] proposed that the failure strength of rock meso-element is closely related to rock failure criteria. In view of the good engineering practice background of the Mohr-Coulomb criteria, the suggestion of Deng [5] and Cao [31] is adopted here to depict the strength of rock meso-element, i. e.

Define Phi

$$F = \frac{E\varepsilon_1\left[(\sigma_1 - \sigma_3) - (\sigma_1 + \sigma_3)\sin\varphi\right]}{\sigma_1 - 2\mu\sigma_3} \quad (26)$$

Discussion of the axial residual strength

Existing studies on rocks mechanics behaviors have shown that the failure process of rocks is the process in which effective elements decrease and failure elements increase. At the same time, macroscopic fracture plane begins to appear slowly and gradually, leading to the gradual reduction of cohesion on the fracture plane and the gradual change of friction strength to a

stable value [33], which also explains the source of rock residual strength.

As mentioned above, through the statistical damage mechanics of rock, it can be considered that the damage meso-element can still bear a certain stress. When rock is completely damaged, the net stress of the damaged elements equals the residual strength. Here the residual strength is used to replace the net stress in the damaged area. Herei , the axial residual strength is obtainable through the equation in Zareifard and Fahimifar [33]

$$\sigma_1^r = 2c_r \cos\varphi_r/(1-\sin\varphi_r) + \sigma^3(1+\sin\varphi_r)/(1-\sin\varphi_r) \quad (27)$$

where ϕ_r is the internal friction angle under residual strength, and c_r is the cohesion under residual strength, linear regression was performed on the experimental data, then these two parameters can be obtainable.

Model Validation

To verify the model proposed in this paper, experimental data for sandstone made by Zeng [35] is adopted here, and the data was also referenced to build the model of Weibull distribution by Cao [31]. The basic physical parameters of the referenced data are given as Young's modulus E=95MPa, Poisson's ratio μ=0.2, the internal friction angle φ=31.064°. The model validation can be divided into two steps. Firstly, the K-S test needs to be carried out on $f_p(x)$. Generally speaking, a good fitting can truly reflect the probability density distribution characteristics of rock strength. Secondly, the maximum entropy model needs to be developed under different confining pressures. The calculated curves by the proposed model are further compared with the experimental curves in Zeng [35] and the simulated curves in Cao [31].

K-S test of the probability distribution function

The K-S test of the maximum entropy density distribution function $f_p(x)$ is required. If

σ_3=5MPa, by Eq. (25), the strength of rock meso-elements can be calculated as (unit: MPa): 0 , 6.99, 17.644, 29.89, 40.87, 65.16, 89.39, 114.77, 136.92, 161.18, 182.00, 201.36, 220.47, 238.05, 252.12, 265.96, 279.23.

The first-order, second-order, third-order origin moments of the above samples are needed to be calculated, then according to Eq. (7), the Lagrange multipliers are computed as: λ_0=4.26732, λ_1=0.02475, λ_2=2.40024×10^{-4}, λ_3=6.24729×10^{-7}. Set the maximum entropy probability density function attained herein as $f(x)$, the corresponding probability distribution function is F(x), and assume H_0: F(x)=F_0(x); Set the empirical probability distribution function as F_n(x), and the statistic D_n can be calculated as

$$D_n = \max_{-\infty<x<+\infty} |F_0(x) - F_n(x)| \tag{28}$$

Here, the sample size n=17, and the significance level α is set at 0.1, the critical value $D_{17,\ 0.10}$ =0.3285, which can be found in the K-S test table [5]. Through Eq. (28), the calculated statistic D_{17}=0.077, obviously $D_{17}<D_{17,\ 0.10}$, therefore, the original hypothesis cannot be rejected. It means that the imitative effect of the maximum entropy probability distribution function obtained is perfect.

Model verification

Under different confining pressures, through the above-discussed calculation method of maximum entropy, the calculations of the Lagrange multipliers are shown in Table 1.

Table 1. The calculated Lagrange multipliers under different confining pressures.

Lagrange multipliers	Confining pressures /MPa			
	0	5	10	20
λ_0	4.45679	4.26732	5.45264	4.57542
λ_1	0.02667	0.02475	0.02547	0.01621

λ_2	-2.053725×10^{-4}	-2.40024×10^{-4}	-2.84668×10^{-5}	-5.58234×10^{-5}
λ_3	3.95647×10^{-7}	6.24729×10^{-7}	-5.45348×10^{-8}	-1.98512×10^{-8}

Through substituting the Lagrange multipliers in Table 1 into Eq. (25), the rock constitutive equation can be determined. Calculated fitting curves are shown in Fig 2.

obtained

Fig 2. Fitting curves of the the maximum entropy model.

move to Fig. 2

very general discussion

From the comparative analysis of the fitting curves in Fig 2, it can be found that the confining pressure influences the axial peak strength of rock and seems to increase in direct proportion. This conclusion is consistent with the traditional rock mechanics. In addition, it can be found that the greater the confining pressure, the greater the residual strength of the rock. The probable reason is that the increase of confining pressure will increase the contact stress between each element or fracture plane inside the rock, and the residual strength will increase when the friction coefficient remains unchanged.

The experimental curves and calculated curves of the Weibull model serve as the control group, the comparison result is shown in Fig 3, and the relative error analysis of the two calculated curves are given in Table 2.

Figure 3. Comparison of experimental and calculated values: (a) σ_2=σ3=0MPa; (b) σ_2=σ3=5MPa; (c) σ_2=σ_3=5MPa; (d) σ_2=σ_3=5MPa.

Table 2. Mean relative errors of Weibull model and proposed model (%).

Model	Confining pressures /MPa				Total mean relative error
	0	5	10	20	

Weibull model	38.80	9.54	7.38	31.74	21.87
Proposed model	7.74	5.64	4.69	23.60	10.41

It is found that the proposed model and the Weibull model introduced by Cao [31] can well reflect the progressive failure phenomenon for rocks before the peak value. In the post-peak curve stage, the axial strength gradually decreases, and the strain continues to grow, this phenomenon belongs to the strain-softening behavior of rocks, and both calculated curves can also well capture this characteristic. At the end of the curve, the axial strength tends to a stable value, it is ostensibly independent of strain, and the stable value is called residual strength. The proposed model considering the residual strength can well describe this phenomenon, while the description ability of the calculated curves in Cao [31] for residual strength is weaker.

Overall, the proposed model can well match the experimental curves, and the overall mean relative error only reaches 10.41%. The average relative error of the fitting results of the maximum entropy model is smaller than the Weibull model under different confining pressures. Although the stress-strain relationship of whole process for rocks can be reflected well by the proposed model, there are still some calculated points which deviate slightly from the experimental curve. Probably there are two reasons: On the one hand, from the statistical standpoint, the damage of rocks is complex, rely on a mathematical distribution function to describe the gradual failure process of rocks seem too idealistic; On the other hand, from the standpoint of the failure mechanism for rocks, in the gradual failure, its physical parameters will inevitably change, and it is difficult to maintain a constant, so further research is needed.

Conclusions

In this research, the maximum entropy distribution function is utilized to reflect the damage characteristics of rock, and a rock constitutive model considering lateral damage is established by using meso-damage statistics theory. Using experimental data, the proposed model is compared with other theoretical models to verify its applicability. The following conclusions are obtained as follows:

1) The expression of the maximum entropy probability density function is deduced to describe the distribution of random variables without any subjective bias. Compared with the classic random distribution, the method can make full use of the sample information without excessively depending on the sample, which has more sufficient mathematical and physical significance.

2) Compared with the experimental curves, the progressive damage process of rock can be well simulated by the proposed model. The total average relative error of the maximum entropy model is only 10.41 %, which is close to the experimental curve. It also reflects that the rock strength is affected by the stress state, the confining pressure has a noticeable influence on the axial peak strength of rock, and there is a directly proportional relationship between them, which is obviously consistent with the traditional rock mechanics. In addition, the proposed model can better reflect the strain-softening behavior for rocks and respond to the residual strength of rocks. In summary, the proposed model is feasible and provides a specific reference for the study of the statistical damage model of rocks.

very general

3) Existing statistical damage constitutive models assume that the strain produced by the undamaged material is consistent with that of the damaged material, based on the assumptions, in the progressive failure process of rock, the physical equation of the material in the damage

is difficult to be determined, or there is a contradiction with the current statistical damage hypothesis, which requires further research and discussion.

Author Contributions

Conceptualization: Xiaoming Li, Mingwu Wang, Fengqiang Shen.

Data curation: Xiaoming Li, Hongfei Zhang.

Formal analysis: Mingwu Wang.

Investigation: Fengqiang Shen, Hongfei Zhang.

Methodology: Xiaoming Li.

Resources: Hongfei Zhang.

Supervision: Fengqiang Shen.

Validation: Xiaoming Li.

Writing-original draft preparation: Xiaoming Li.

Writing-review and editing: Mingwu Wang.

Acknowledgments

Financial support partially provided by the National Natural Sciences Foundation of China (No. 41172274) is gratefully acknowledged.

References

1. Cook, N.G.W. Cook. The failure of rock. *Int. J. Rock. Mech. Sci.* 1965,2,389–403.
2. Barla, G; Barla, M; Debernardi, D. New triaxial apparatus for rocks. *Rock Mechanics and Rock Engineering*. 2010, 43, 225–230. https:doi:10.1007/s00603- 009-0076-7.

3. Chen, S; Qia, C; Ye, Q; Khan, M.U. Comparative study on three dimensional statistical damage constitutive modified model of rock based on power function and Weibull distribution. *Environ Earth Sci*. 2018, 77, 108. https://doi.org/10.1007/s 1266 5-01 8-7297-6.
4. Desai, C.S; Toth, J. Disturbed state constitutive modeling based on stress-strain and nondestructive behavior. *International Journal of Solids and Structures*. 1996, 33, 1609–1650. https://doi:10.1016/0020-7683(95)00115-8.
5. Deng, J; Gu, D. On a statistical damage constitutive model for rock materials. *Comput Geosci*. 2012, 37, 122–128. https://d oi.org/10.1016/j. cageo.2010.05.018.
6. Fang, X; Xu. J; Wang, P. Compressive failure characteristics of yellow sandstone subjected to the coupling effects of chemical corrosion and repeated freezing and thawing. *Eng Geol*. 2018, 233, 160–171. https://doi.org/10.1016/j.enggeo.2017.12.014.
7. Martin, C.D; Chandler, N.A. The progressive fracture of Lacdu Bonnet granite. *International Journal of Rock Mechanics and Mining Sciences & Geomechanics Abstracts*. 1994, 31, 643–659. https://doi:10.1016/0148-9062(94)90005-1
8. Krajcinovic, D; MSilva, M.A.D. Statistical aspects of the continuous damage theory. *International Journal of Solids and Structures*. 1982, 18, 551–562. https://doi.org/10.101 6/002 0-7683(82)90039-7.
9. Basu, B.D; Tiwari, D; Kundu, D; Prasad, R. Is Weibull distribution the most appropriate statistical strength distribution for brittle materials. *Ceram. Int.* 2007, 35, 237–246. https:// doi.org/10.1016/j.ceramint.2007.10.003.
10. Li, X; Cao, W.G; Su, Y.H. A statistical damage constitutive model for softening behavior of rocks. *Eng. Geol*. 2012, 144, 1–17. https://doi.org/10.1016/j.enggeo.2012.05.005.
11. Liu, H.Y; Su, T.M. A dynamic damage constitutive model for a rock mass with non-persistent joints under uniaxial compression. *Mech. Res. Commun*. 2016, 77, 12–20. https://doi.org/10.10 16/j.mech rescom.2016.08.006.

12. Mortazavi, A; Molladavoodi, H. A numerical investigation of brittle rock damage model in deep underground openings. *Eng. Fract. Mech*. 2012, 90, 101–120. https://doi.org/10.1016/j.e ngfracmech.2012.04.024.
13. Wang, Z.L; Li, Y.G; Wang, J.G. A damage-softening statistical constitutive model considering rock residual strength. *Comput. Geosci*. 2007, 33, 1–9. https://doi.org/10.1016/j.cageo.2006.02 .011.
14. Zhou, J.W; Xu, W.Y; Yang, X.G. A microcrack damage model for brittle rocks under uniaxial compression. *Mech. Res. Commun*. 2010, 37, 399–405. https://doi.org/10.1016/j.mec h-rescom.2 010.05.001.
15. Xu, W.Y; Wei, L.D. Study on statistical damage constitutive mode of rock. *Chinese Journal of Rock Mechanics and Engineering.* 2002, 21, 787–791.
16. Xu, X.L; Karakus, M; Gao, F; Zhang, Z.Z. Thermal damage constitutive model for rock considering damage threshold and residual strength. *J. Cent. South. Univ*. 2018, 25, 2523–2536. https://doi.org/10.1007/s11771-018-3933-2.
17. Zhu, L.J; Xu, X.L; Cao, X.J; Chen, S.Y. Statistical constitutive model of thermal damage for deep rock considering initial compaction stage and residual strength. *Mathematical Problems in Engineering*. 2019, 9035396. https://doi.org/10.115 5/201 9/9035396.
18. Cao, W.G; Tan, X; Zhang, C; He, M. Constitutive model to simulate full deformation and failure process for rocks considering initial compression and residual strength behaviors. *Canadian Geotechnical Journal*. 2019, 56, 649–661. https://do i:10.1 139/cgj-2018-0178.
19. Tan, C; Yuan, M.D; Shi, Y.S; Zhou, B.S. Statistical damage constitutive model for cemented sand considering the residual strength and initial compaction phase. *Advances in Civil Engineering*. 2018, 1410893, 1–9. https://doi.org/10.1155/2018/1410893.
20. Zhao, H.Z; Shi, C.J; Zhao, M.H; Li, X.B. Statistical damage constitutive model for rocks considering residual strength. *Int. J. Geomech*. 2016, 17, 04016033. https://doi. 10.1061/(ASCE)GM.1943-5622.0000680.

21. Jiang, H.B; Li, K; Hou, X.B. Statistical damage model of rocks reflecting strain softening considering the influences of both damage threshold and residual strength. *Arabian Journal of Geosciences*. 2020, 13, 286–294. https://doi.org/10.1007/s125 17-020-5270-7.
22. Lin, Y; Zhou, K.P; Gao, F; Li, J. Damage evolution behavior and constitutive model of sandstone subjected to chemical corrosion. *Bulletin of Engineering Geology and the Environment*. 2019, 78, 5991–6002. https://doi.org/10.1007/s10064-019-01500-7.
23. Danzer, R. Some notes on the correlation between fracture and defect statistics: are Weibull statistics valid for very small specimens?. *J. Eur. Ceram. Soc*. 2016, 26, 3043–3049. https://doi.org/10.1016/j.jeurceramsoc.2005.08.021.
24. Basu, B; Tiwari, D; Prasad, R. Is Weibull distribution the most appropriate statistical strength distribution for brittle materials?. Ceram. Int 2009, 35, 237–246. https://doi.org/10.1016/j.ceramint.2007.10.003.
25. Danzer, R; Supancic, P; Pascual, J; Lube, T. Fracture statistics of ceramics-Weibull statistics and deviations from Weibull statistics. *Eng. Fract. Mech*. 2007, 74, 2919–2932. https://doi.org/10.1016/j.engfracmech.2006.05.028.
26. Todinov, MT. Is Weibull distribution the correct model for predicting probability of failure initiated by non-interacting flflaws? *Int. J. Solids. Struct.* 2009, 46, 887–901. https://doi.org/10.1016/j.ijsolstr.2008.09.033.
27. Jaynes, E.T. Information theory and statistical mechanics. *Phys Rev*. 1957,106, 620.
28. Jaynes, E.T. Information theory and statistical mechanics II. *Phys Rev*. 1957,108, 171.
29. Shannon, C.E. A mathematical theory of communication. *Bell. Sys.t Techn. J*. 1948, 27, 623–656.
30. Lemaitre, J; Chaboche, J.L. Mechanics of solid materials. Cambridge University Press, Cambridge, U.K, 1990.
31. Cao, W.G; Zhao, M.H; Liu, C.X. Study on rectified method of Mohr-Coulomb strength criterion for rock based on statistical damage theory. *Chinese Journal of Rock Mechanics & Engineering*. 2005, 24, 2403–2408.

32. Pariseau, W.G. Fitting failure criteria to laboratory strength tests. *Int. J. Rock Mech. Min. Sci.* 2007,44, 637–646. https://doi.org /10.1016/j.ijrmms.2006.09.006.
33. Menendez, B; Zhu, W; Wong, T. Micromechanics of brittle faulting and cataclastic flflow in Berea sandstone. *J. Struct. Geol.* 1996, 18, 1–16.
34. MZareifard, M; Fahimifar, A. Elastic–brittle–plastic analysis of circular deep underwater cavities in a Mohr-Coulomb rock mass considering seepage forces. *Int. J. Geomech*. 2014, 15, 04014077. https://doi:10.1061/(ASCE)GM.1943-5622.0000400.
35. Zeng, Y.W; Zhao, Z.Y; Zhu, Y.W. Bifurcation analysis on failure forms of rock material. *Journal of Rock Mechanics and Engineering*. 2002, 21, 948–952.

Figure
Click here to access/download;Figure;Figure.docx

Fig. 1

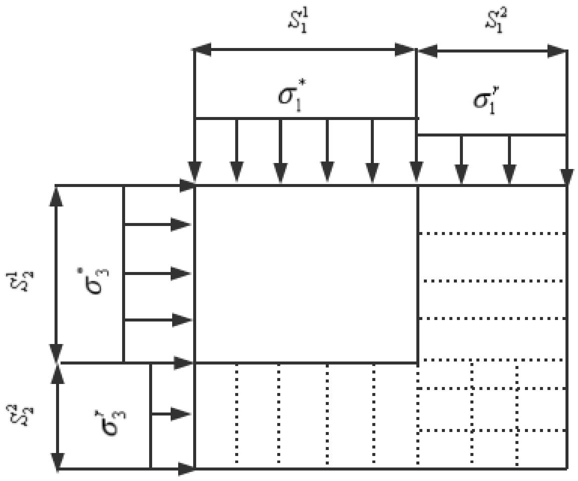

Fig. 2

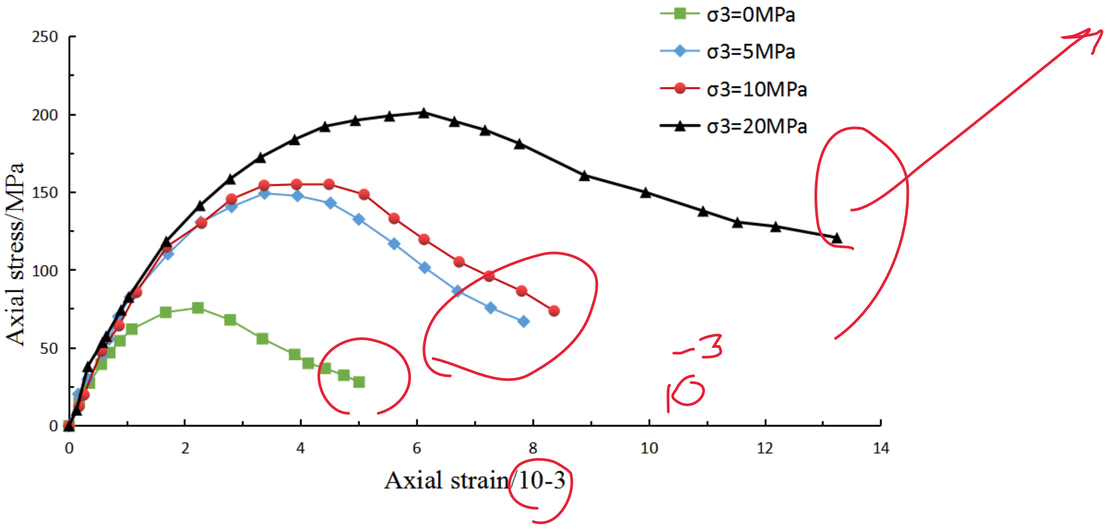

How the authors defined this part of the curve as "residual strength". this should be defined very carefully.

Fig. 3

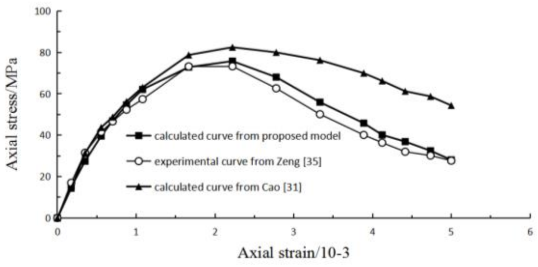

(a) σ_2=σ_3=0MPa

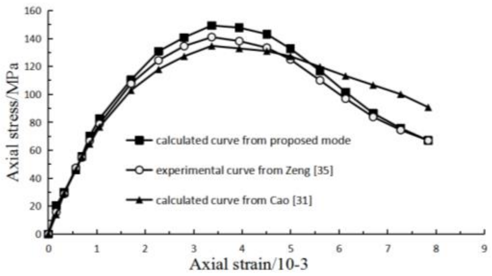

(b) σ_2=σ_3=5MPa

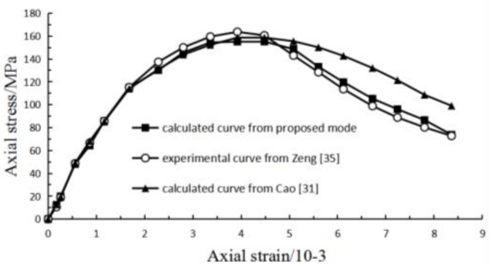

(c) σ_2=σ_3=5MPa

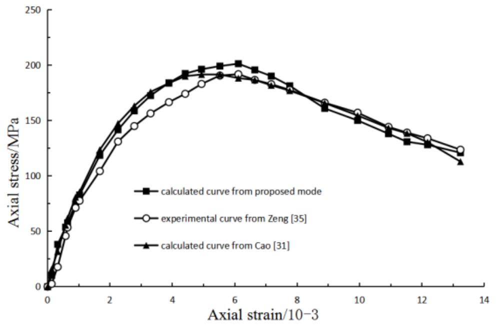

(d) σ_2=σ_3=5MPa
