# Supplementary material for: Modeling for strain-softening rocks with lateral damage based on statistical physics
Source: PLoS One. 2023 Mar 30;18(3):e0283313. doi: 10.1371/journal.pone.0283313 (PMC10062584; doi:10.1371/journal.pone.0283313)
Supplement: Attachment — Submitted filename: Review comments.doc [file pone.0283313.s002.doc]

In this manuscript, a new statistical constitutive model considering the lateral damage is proposed based on the meso-damage statistics theory, and its constitutive relation equation is deduced. Furthermore, using experimental data, the proposed model is verified by comparing the Weibull model.

The paper is well organized, however, there are some modifications before publication in PLOS ONE.

1.It is suggested that Eq. (14) is written as following:

$\sigma^{*}\text{=}\frac{\sigma}{\text{1-}D}=\frac{E\varepsilon}{\text{1-}D}$(14)

2. Please check whether Eq. (17) is correct?

3. The names of Fig 3b,3c and 3d are wrong, please modify it.

4. It is suggested that more examples are used to validate the validity of the proposed model.
